# Financing the transition: a roadmap for sustaining disease control in low- and middle-income countries after the 2025 contraction in development assistance for health

**DOI:** 10.3389/frhs.2026.1902705

**Published:** 2026-08-19

**Authors:** Shyamkumar Sriram

**Affiliations:** Department of Rehabilitation and Health Services, College of Public Affairs and Health Sciences, University of North Texas, Denton, TX, United States

**Keywords:** debt-for-health swaps, development assistance for health, domestic resource mobilization, fiscal sovereignty, health taxes, Lusaka agenda, pooled procurement, progressive taxation

## Abstract

In 2025, development assistance for health fell by about 21% in a single year, driven largely by a 67% reduction in United States financing, disrupting HIV, tuberculosis, malaria, immunization and maternal health programs across low- and middle-income countries. Modelling indicates millions of avoidable deaths by 2030 if the resulting gap is not filled, yet most recipient governments have not reprioritized budgets or raised domestic revenue in response. This brief argues that the contraction is structural rather than cyclical and sets out a sequenced transition-financing roadmap. It proposes phased, country-led actions: protect frontline services immediately; mobilize domestic revenue through progressive as well as consumption taxes and restructure debt over the medium term; and embed efficiency and pooled procurement under the Lusaka Agenda, without allowing efficiency to substitute for adequate public financing.

## Introduction

For two decades, external financing carried much of the fight against communicable disease in poorer countries, and the United States carried more of it than anyone else. In 2025 that support fell away with little warning. Development assistance for health dropped by about 21% in a single year, most of it traceable to a 67% cut in United States funding worth more than US$9 billion (1). The agency that had delivered much of that aid, the United States Agency for International Development, was dissolved; of some 770 global health awards, roughly four in five were cancelled, stranding about US$12.7 billion in commitments (2).

Washington was not alone. The United Kingdom, France and Germany have all cut their official development assistance for the first time in decades, with further reductions due to bite in 2026 (3). The wider picture is just as stark: the OECD projects official development assistance falling by 9–17% in 2025, and by 16–28% for sub-Saharan Africa, with health funding potentially dropping as much as 60% from its 2022 peak (3).

**Figure 1:**
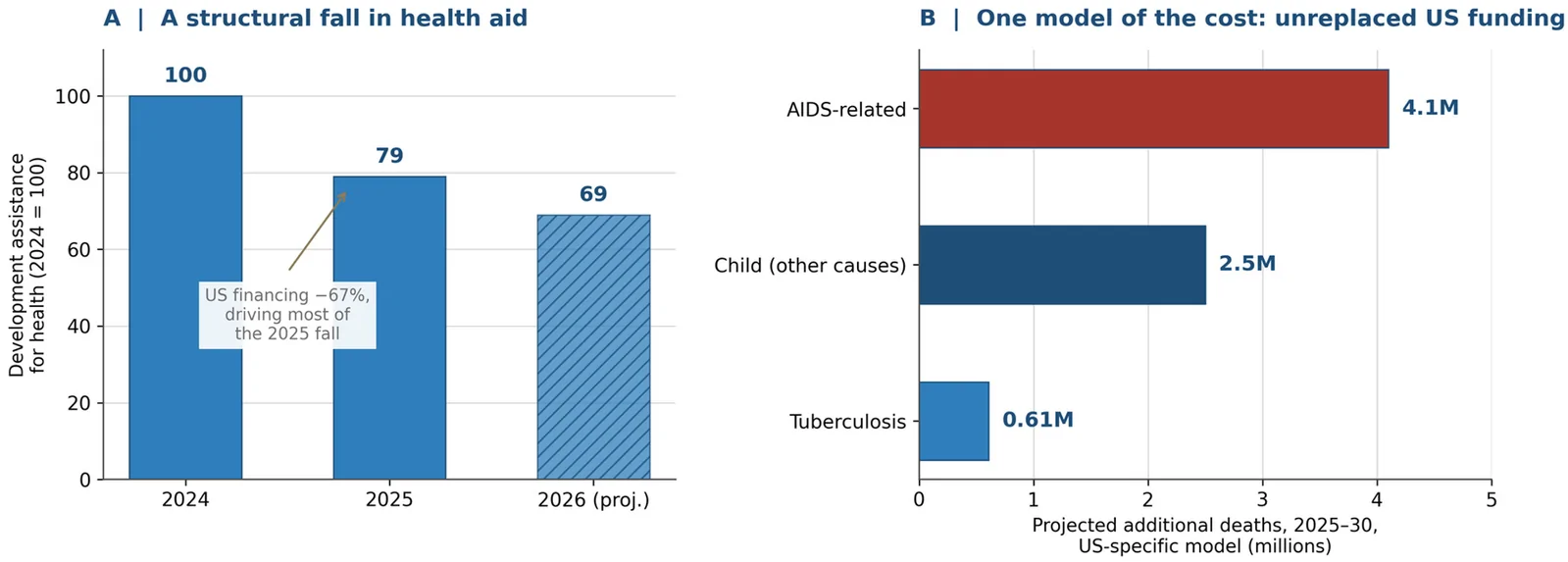
The scale of the shock and the stakes. Panel **(A)**: development assistance for health relative to 2024, with a 21% fall in 2025 and further decline expected in 2026. Panel **(B)**: projected additional deaths between 2025 and 2030 under an unreplaced cessation of United States assistance, by cause, from a disease-specific modelling study covering HIV, tuberculosis and child health (6). Panel **(B)** is not comparable with, and should not be added to, the ODA-wide projections reported in the text (4), which model all official development assistance across 93 recipient countries. Sources: funding data (1, 3); mortality projections (6).

What an unfilled gap costs is measured in lives, and the available models estimate that cost at different scopes (Figure 1). At the broadest scope, a retrospective evaluation and forecasting analysis of official development assistance across 93 recipient countries projects 9.4 million additional deaths by 2030 (95% UI 6.2–12.6 million), including 2.5 million among children under five, under a mild defunding scenario in which the average rate of decline observed in 2024 and 2025 continues; under a severe scenario in which cuts deepen and stabilize at a lower baseline through 2030, the projection rises to 22.6 million additional deaths (95% UI 16.3–29.3 million), including 5.4 million among children under five (4). Narrower in scope, an evaluation of two decades of USAID-supported programs across 133 countries, which associates that funding with 91 million deaths averted between 2001 and 2021, projects 14.1 million additional deaths by 2030 (95% UI 8.5–19.7 million), including 4.5 million among children under five, if support for former USAID programs remains largely halted (5). Narrower still, a disease-specific modelling study of an unreplaced cessation of United States assistance puts the toll at roughly 4.1 million additional AIDS-related deaths, 607,000 additional tuberculosis deaths and 2.5 million additional child deaths between 2025 and 2030 (6). For the first year alone, a budget-based estimate using the decline in United States outlays suggests 500,000 to one million deaths in 2025 (7).

These figures are not additive and should not be read as competing estimates of the same quantity. They differ in the funding streams modelled, from all official development assistance to a single donor agency to three disease programs; in the countries covered, 93, 133 or a subset; and in the counterfactual assumed about replacement by other donors and by domestic budgets. All are model-based projections with wide uncertainty intervals, and all are conditional on the cuts persisting. What they agree on is direction and order of magnitude: on current trends the shortfall costs millions of lives this decade, and the range across scopes is a reason to act early rather than a reason to discount the estimates.

These losses land on systems that were already fragile. In the poorest countries, roughly a quarter of health spending comes from abroad (8), and many governments now pay more to service their debts than they spend on health and education combined (10). Households were already absorbing what public financing did not: 2.1 billion people faced financial hardship from out-of-pocket health payments in 2022, and 1.6 billion were pushed into poverty or further into it (9). The 2025 cuts did not so much create that vulnerability as expose it.

So far, the response has been muted. A review of sub-Saharan budget statements found that, for the most part, governments have not raised taxes or shifted spending to cover the shortfall, and health budgets are set to shrink in 2026 before recovering (11). On this evidence, waiting for the aid to return is a poor plan.

This brief treats the contraction as structural rather than temporary and sets out a transition-financing roadmap to match. It separates what can be done now from what belongs to the next one to three years, and to the three to five years beyond that, and it assigns the work across national governments, global health initiatives (GHIs) and multilateral institutions, with the aim of turning a funding shock into fiscal and health-system sovereignty. Self-reliance is used here in that sense, meaning the capacity to decide what is financed and for whom and to raise the revenue to do it, rather than simply a smaller external share of the health budget. A structural diagnosis calls for a response that addresses the terms of financing, not only its sources.

## Policy options and implications

Five broad responses are open to exposed governments. They are not so much alternatives as ingredients; the argument here is that they have to be combined, and sequenced to fit each country's fiscal room, debt position and reliance on aid.

### Option 1 – wait for donor funding to return

Holding programs steady in the hope that money comes back is the least disruptive path in the moment, and the least defensible over time. The cuts are driven by lasting pressures, among them competing demands for defense spending, and further falls are already forecast. Even a partial recovery would leave deep holes: restoring money for HIV treatment but not prevention would still allow close to a million new infections by 2030 (6). To wait is to risk programs collapsing and diseases returning.

### Option 2 – reprioritize and accelerate co-financing

Shifting money within health budgets, speeding up co-financing pledges to the Global Fund and Gavi, and protecting the supply of essentials, from antiretrovirals and anti-tuberculosis medicines to bed nets and vaccines, can hold the line for a while. This buys time rather than solving the problem, and it strains quickly where fiscal space is thin. Reprioritization is also a distributive decision rather than a technical one, since it determines whose care is protected when budgets contract, and the criteria should be stated rather than left to bargaining strength. Four principles are proposed: preserve essential public health functions such as surveillance, outbreak response and vector control; protect primary and preventive care rather than defending tertiary budgets by default; shield the populations already facing the greatest barriers to care; and do not close the gap by shifting costs onto households through user fees or informal payments, which converts a fiscal problem into an impoverishing one (9).

### Option 3 – mobilize domestic revenue

Taxes on tobacco, alcohol and sugary drinks raise revenue and curb non-communicable disease at the same time, while broader social health insurance and a wider tax base can enlarge the pool over time (12). The gains are real but gradual, and in the poorest, most aid-dependent countries they cannot close the gap quickly. Excise taxes also concentrate the burden of adjustment on consumption, so their distributive effect depends on how the revenue is spent and on what else is taxed alongside them. A response proportionate to a structural shock should widen the direct tax base as well: taxation of wealth, high incomes, capital gains and luxury consumption, together with action on the profit shifting and offshore wealth holding that cost countries an estimated US$492 billion a year in forgone revenue, around two thirds of it through multinational profit shifting (13). For many aid-dependent countries the binding constraint is less the absence of a tax instrument than the absence of effective taxing rights over income generated within their borders, which is why domestic revenue reform and international tax cooperation belong in the same conversation.

### Option 4 – restructure debt for health

Debt-for-health swaps let heavily indebted countries turn debt service into health spending, which matters most where repayments already crowd out health (12). They help at the margin. Since 2007 the Global Fund's Debt2Health mechanism has completed 14 transactions involving three creditor countries and 11 debtor countries, converting close to US$500 million of bilateral debt into about US$330 million of health financing; agreements signed in 2024 converted €29 million of Mongolian and €75 million of Indonesian debt, and an earlier Australia-Indonesia swap financed tuberculosis treatment for an additional 115,000 people (14). Set against a fall of more than US$9 billion in United States funding in a single year, that is roughly two decades of global debt-swap activity against a fraction of one year's shortfall. The instrument should therefore be understood as a response to a symptom of a larger condition. The binding constraint is the architecture of sovereign debt itself: a record US$921 billion in net interest paid by developing countries in 2024, borrowing costs several times those available to wealthier states, and the absence of an orderly workout mechanism (10). Swaps operate within those terms; restructuring, refinancing on concessional terms and reform of the resolution process change them, and the first should not be offered as a substitute for the second. The Global Financing Facility offers a cautionary lesson, though: loan-heavy arrangements have sometimes replaced public spending rather than adding to it, and tightened the fiscal squeeze (15). Debt instruments should supplement public money, never stand in for it.

### Option 5 – pursue efficiency, integration and pooled procurement

The Lusaka Agenda for Sustainable Health Financing sets out five shifts: less fragmented aid, stronger country ownership, vertical programs folded into primary care, joint procurement to cut the price of medicines, and shared accountability (16). It is gaining traction. In May 2025 the World Health Assembly passed its first health-financing resolution in fifteen years, citing the Agenda, as did a resolution at the United Nations General Assembly (17). Efficiency and integration free up resources without new taxes and soften the blow of single-disease funding shocks, although both take time and political effort.

Pooled procurement has a long empirical record. The Organisation of Eastern Caribbean States Pharmaceutical Procurement Service pools purchasing for nine member states through a revolving fund held at the Eastern Caribbean Central Bank, to which each country commits a share of its pharmaceutical budget in advance; it is one of the mechanisms from which reviews report average medicine price reductions of about 15% relative to individual purchasing, alongside the PAHO Strategic Fund in Latin America and the Caribbean (18). The precondition is visible in the same record: savings materialize where countries can forecast demand, commit budget and pay on time, and prices can rise rather than fall where less creditworthy members join a pool without those capacities.

Efficiency needs one qualification. It protects scarce resources; it does not create the fiscal space that adequate public financing would provide, and a sustained call to do more with less can normalize the chronic underfinancing it is meant to mitigate. Integration and joint procurement should therefore accompany binding commitments to raise public spending on health, not stand in for them. The test of a successful transition is not only whether systems become cheaper to run, but whether they become adequately and equitably financed.

## What stands in the way

Options 3–5 are constrained less by technical design than by fiscal space, institutional capacity and the politics of taxation.

### Adjustment programs and conditionality

Where a fund-supported program is in place, fiscal targets and public wage-bill ceilings shape what health ministries can spend and whom they can hire. In 16 West African countries, each additional binding IMF condition was associated with a 0.248% reduction in government health expenditure per capita (95% CI −0.435 to −0.060), with constrained fiscal space, wage-bill ceilings limiting the hiring and retention of doctors and nurses, and budget execution difficulties identified as the operative pathways (19). This is why a health-protective spending floor is assigned in the roadmap to the multilateral institutions: it is a design feature of the adjustment program, not something a health ministry can secure alone.

### Legislative lag and industry resistance on health taxes

Excise reform requires primary legislation and survives only if it outlasts the government that passed it. As of 2025 only about 15% of the world's population was covered by tobacco taxes at the level WHO regards as best practice, and 40 countries had not implemented a single MPOWER measure at that level (20). Progressive direct taxes face the same problem with stronger opposition, which is why both belong in the 1–3 year horizon rather than the stabilization phase.

### Administrative capacity for prepayment

Extending contributory insurance to informal workers is an administrative problem before it is a financing one. Two decades after Ghana legislated its National Health Insurance Scheme, active enrolment has hovered around 40% of the population, with informal-sector workers among the hardest to recruit and retain (21). Where identification, collection and renewal systems are weak, tax-financed expansion of a defined benefit package will usually add covered lives faster than premium-based enrolment.

No single lever is enough. Hoping for restoration is imprudent; emergency measures only steady the ship; domestic revenue and debt swaps are powerful but slow and bounded; and efficiency and integration, while essential, are structural and gradual. What works is a sequence, which is how the recommendations below are arranged.

## Actionable recommendations

The roadmap below (Table 1) arranges action across three horizons and three sets of actors. The principle is sequence: steady the frontline first, build sustainable revenue and restructure debt next, and embed efficiency and country-led financing for the long run. Table 2 sets the main financing instruments against their likely effect, the time they take and their principal risk.

1. **Stabilize (0–12 months).** Governments should review spending quickly, ringfence the budget for life-saving commodities and frontline staff, and honor or bring forward their Global Fund and Gavi co-financing. Where cuts are unavoidable, they should follow the reprioritization principles set out in Option 2 rather than fall where resistance is weakest. Donors and GHIs should keep bridging finance flowing, make good on existing pledges and avoid the abrupt stops that leave patients stranded. The International Monetary Fund and World Bank should hold health spending above a defined floor within any adjustment program.
2. **Mobilize (1–3 years).** Governments should introduce or raise health taxes and earmark a share for health, broaden direct and progressive taxation alongside them, widen social insurance and prepayment, and negotiate debt-for-health swaps where debt distress is severe, taking care that they genuinely add to public spending. This is also the moment to begin folding vertical programs into primary care and to join pooled-procurement platforms.
3. **Transform (3–5 years).** Governments should push health spending up as a share of both gross domestic product and the total budget. GHIs should move from buying commodities to strengthening systems, with far less fragmentation, in line with the Lusaka Agenda. Countries and regional bodies should invest in resilient procurement and regional manufacturing so that supply no longer depends on distant chains. Multilateral institutions and creditor governments should pursue debt restructuring and international tax cooperation, without which domestic revenue effort is bounded by terms set elsewhere.
4. **Protect equity and accountability (throughout).** Transparent expenditure tracking, mutual accountability between governments and partners, and explicit protection for the poorest should run through every phase, so that the shift in financing does not fall hardest on those least able to bear it. Progress should be judged on financial protection and coverage among the poorest, not on aggregate spending alone.

**Table 1:** Phased transition-financing roadmap for sustaining disease control after the 2025 contraction in development assistance for health.

Phase	Horizon	Government actions	Donor/GHI actions	Multilateral actions
Stabilize	0–12 months	Rapid expenditure review; ringfence commodities and workforce; meet co-financing obligations	Bridge financing; honor pledges; no abrupt termination; align to national plans	Health-protective spending floors within fiscal programs
Mobilize	1–3 years	Health taxes; expand social insurance; debt-for-health swaps with additionality; begin PHC integration; broaden progressive direct taxation	Shift toward predictable, pooled support; co-fund procurement platforms	Concessional finance; debt restructuring support; technical assistance
Transform	3–5 years	Raise government health spending as share of GDP and budget; resilient procurement	Transition from commodities to system strengthening; cut fragmentation	Sustain country-led financing; support regional manufacturing; debt restructuring and international tax cooperation

GHI, global health initiative; PHC, primary health care; GDP, gross domestic product.

**Table 2:** Domestic and structural financing instruments, with indicative effects, time horizons principal risks and documented country or regional experience.

Financing instrument	Indicative effect	Time to materialize	Principal risk	Documented experience
Health taxes (tobacco, alcohol, sugar)	New revenue plus reduced NCD burden	Short to medium	Political resistance; regressivity if poorly designed	Only about 15% of the world's population covered by tobacco taxes at best-practice level as of 2025 (20)
Social health insurance/prepayment	Broader, pooled financing base	Medium	Slow enrolment; informal-sector coverage gaps	Ghana NHIS active enrolment around 40% of the population two decades after enactment (21)
Debt-for-health swaps	Redirects debt service to health	Medium	Limited scale; substitution for public spending	Global Fund Debt2Health: about US$330 million for health across 11 countries since 2007 (14)
Pooled procurement	Lower commodity unit costs	Short to medium	Requires governance and coordination capacity	OECS Pharmaceutical Procurement Service and PAHO Strategic Fund; average price reductions of about 15% reported across reviewed mechanisms (18)
Progressive direct taxes and anti-avoidance measures	Broader and more equitable revenue base; addresses a source of the fiscal constraint	Medium to long	Requires taxing rights, administrative capacity and international cooperation	About US$492 billion lost annually to cross-border corporate and individual tax abuse (13)

NCD, non-communicable disease; NHIS, National Health Insurance Scheme; OECS, Organisation of Eastern Caribbean States; PAHO, Pan American Health Organization.

## Conclusions

The 2025 contraction is a turning point, not a passing dip. Waiting for donors to return will cost lives and unravel decades of progress against HIV, tuberculosis, malaria and vaccine-preventable disease. Handled differently, the same shock can push countries towards the domestically financed, country-led systems the Lusaka Agenda imagines, provided governments and their partners move in order: steady services now, raise revenue and restructure debt over the medium term, and build efficiency and joint procurement for the long term. A structural shock, though, is not answered by instruments alone. Who is taxed, whose care is protected when budgets contract, and on whose terms debt is serviced are distributive questions, and leaving them implicit is itself a choice. The standard for this transition is sovereignty over what is financed and for whom, not simply a smaller external share of the budget. The path is demanding, but it is walkable, and the alternative is the return of diseases the world already knows how to hold back.
